# Improving the nutrient quality of foods and beverages using product specific standards for positive nutrients and ingredients will help to increase mean population intakes toward dietary guidelines

**DOI:** 10.3389/fnut.2023.1292231

**Published:** 2023-12-06

**Authors:** Mariska Dötsch-Klerk, Sara Carvalho, Corrine F. Lawrence, Julie I. Willems

**Affiliations:** Unilever Foods Innovation Centre, Wageningen, Netherlands

**Keywords:** nutrient profiling, food quality, reformulation, modeling, positive nutrition

## Abstract

**Background:**

A shift toward more sustainable diets, rich in plant-based foods and with fewer animal-derived foods, is needed and will lead to improved health and environmental benefits. Food industry needs to play a part and broaden the scope of product reformulation beyond the reduction of nutrients to limit to increasing ingredients and nutrients in line with dietary recommendations for a healthy sustainable diet.

**Methods:**

The Positive Nutrition Standards (PNS) were defined to increase the consumption of recommended ingredients and nutrients. The PNS were set by translating WHO and Codex guidance into product group standards, considering the role of the product group in the diet. The potential impact of the PNS for vegetables, wholegrain and fibre was modeled using data from the US NHANES 2017–2018 survey, assuming that, foods consumed would be reformulated to meet the standards where relevant.

**Results:**

The modeling showed that application of the PNS could increase mean population intakes by 30% for fibre, by more than 50% for vegetables and even double the intake of wholegrain. However, reformulation alone would not be sufficient to reach recommended intake levels.

**Conclusion:**

The PNS described in this paper can help to increase intakes of relevant positive nutrients and ingredients. However, a multistakeholder approach is needed to encourage consumers to make additionally required dietary shifts to meet the recommendations for positive nutrients and ingredients.

## 1 Introduction

Diets are a key link between population health and environmental sustainability, as unhealthy diets, the rise of non-communicable diseases, and the declining health of the planet are highly intertwined (1). The Global Burden of Disease study has shown that high intakes of sodium, trans fat, sugar-based beverages and processed meat, and low intakes of fruits, vegetables, legumes, wholegrain, nuts, seeds, poly unsaturated fatty acids including essential the omega-3 and omega-6 fatty acids, fibre, and calcium are dietary factors contributing most to disability-adjusted life-years (DALYs) and mortality (2). In addition, food production is a major driver of global environmental footprints (1, 3).

A shift toward more sustainable diets, rich in plant-based foods and fewer animal-derived foods, is needed and will lead to improved health and environmental benefits (3). There have been several efforts to establish guiding principles to define healthy sustainable diets and to integrate this into dietary guidance (3, 4) and food policy (5). These existing sustainable nutrition frameworks and proposed diets focus almost exclusively on whole foods, neglecting the significant contribution of processed and packaged foods to dietary consumption (6). Most packaged foods are considered ultra-processed foods (UPF) in the widely cited NOVA classification (7). Although many epidemiological studies have shown an association between UPF consumption and health risks, these studies were mainly observational studies (8–10), and there is only limited understanding of the mechanism(s) of action (10, 11). The scientific community is still debating the implications of UPFs given their broad definition and heterogeneity (8, 9, 12, 13). Processed foods can vary considerably in their nutritional quality and impact on equity and environment (9, 10). Moreover, processing can add to convenience, improve food safety, extend shelf-life, and offers opportunities for fortification or making nutrients more bioavailable (9–11, 14). Processed foods have long been recognized as a contributor to food and nutrition security (9, 10). Rather than elimination, reformation will have a more meaningful impact on improving the nutritional quality and health on a population level (11). Therefore, processed and packaged foods are a useful part of the solution to make healthy, sustainable dietary patterns achievable and accessible to all (9, 10, 14).

The past decades food industry has mainly focused on the reduction of nutrients to limit such as saturated fat, trans fat, sodium and sugar with the aim to decrease intakes of these nutrients in line with WHO’s Global Strategy on Diet, Physical Activity and Health in 2003 (15) and Global Action Plan for the Prevention and Control of Noncommunicable Diseases 2013–2020 (16). However, considering the high prevalence of malnutrition and micronutrient deficiencies (2, 3, 17) and the potential impact of the move toward more sustainable plant-forward diets, it is important for food industry to broaden the scope beyond the reduction of nutrients to limit to also increase amounts of ingredients and nutrients with a positive health impact (9, 14, 18).

In absence of appropriate guidance or standards for positive nutrients and ingredients to steer food reformulation and innovation, we defined the Positive Nutrition Standards (PNS). The PNS are a set of standards which aim to increase nutrients and ingredients that consumers should eat more of, for human but also planetary health. The current paper describes the selection of the nutrients and ingredients as well as the rationales behind the standards. As proof of principle, modeling analysis was performed using data from the US NHANES 2017–2018 food consumption survey (19), to estimate to what extent reformulation toward the standards could help to increase mean population intakes of vegetables, wholegrain and fibre.

## 2 Materials and methods

### 2.1 Setting the standards

#### 2.1.1 Principles

**Figure 1:**
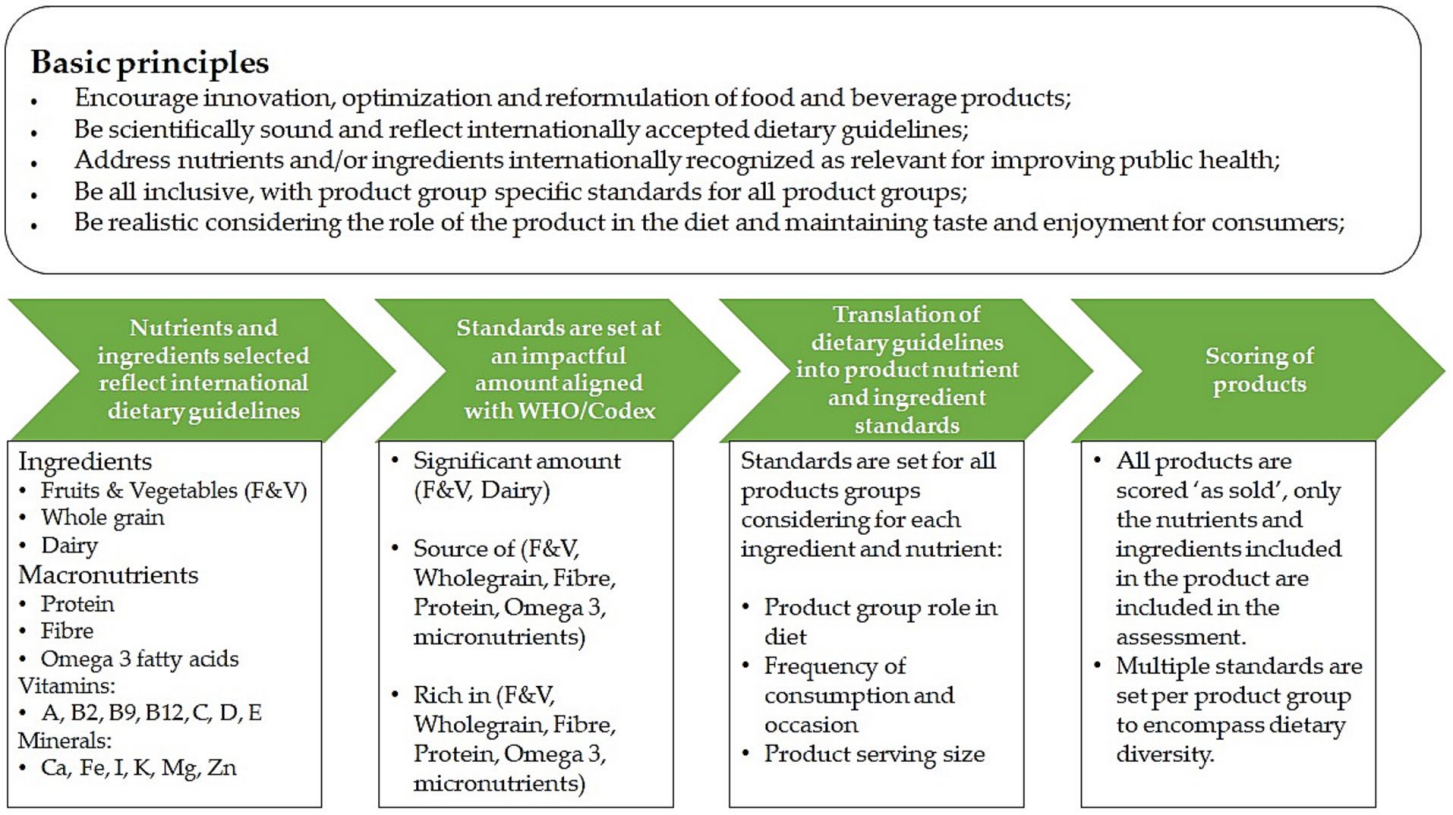
Schematic approach applied for setting the positive nutrition standards.

The positive nutrition standards were set by taking five global principles into account (Figure 1), similar to the standards for saturated fat, transfat, sodium and sugars described in an earlier paper (20). For the Positive Nutrition Standards this means that they reflect dietary guidelines, are set at an impactful amount, encourage the transition toward more sustainable healthy plant-forward diets while also considering the potential implications of this move on nutrient intakes.

Details on the selection of the ingredients and nutrient as well as the approach taken for setting the standards is provided in the next paragraphs.

#### 2.1.2 Type of nutrient profiling method

The standards follow a product-group based approach, which considers the role of the product in the diet, with one absolute standard per ingredient or nutrient by product group rather than using a generic scoring algorithm with multiple thresholds. The product groups were defined in alignment with the company’s current product portfolio. They include a variety of positives per product group, in order to suit a variety of dietary needs.

#### 2.1.3 Selection of ingredients and nutrients

Positive ingredients were selected based on inter(national) dietary guidelines with the aim to promote sustainable healthy plant-forward diets (3, 4, 21, 22). These are the ingredients that are recommended ‘to eat more of’ and that should be promoted in peoples’ diets. Positive nutrients included the internationally recognized micronutrients most relevant for public health (17, 23) and selection of additional nutrients was primarily based on the recent most comprehensive systematic review investigating and comparing nutrient intakes in meat-based, vegetarian and vegan diet pattens in adults in different regions (24). More details on the selection of ingredients and nutrients are provided below and in the flowchart presented in the supplementary material.

##### 2.1.3.1 Ingredients

In 2019, Eat-Lancet researchers looked at current food intakes across regions. The results showed that red meat and starchy vegetables are over-consumed in most regions in comparison to dietary recommendations. Conversely vegetables, fruits, legumes, wholegrains and nuts are underconsumed in every region (3). To provide guidance for the necessary shifts, the Eat-Lancet Commission proposed the Planetary Health Diet, recommending increased consumption of plant-based foods – including fruits, vegetables, nuts, seeds and whole grains – while in many settings substantially decreasing animal source foods.

Similar guidance is provided by FAO/WHO Sustainable Healthy Diets Guiding Principles that recommends to “Eat an abundance and variety of fruits and vegetables and include wholegrains, legumes and nuts; include moderate amounts of eggs, dairy, poultry and fish; and small amounts of red meat.; adequate (but not exceeding needs) in energy and nutrients for growth and development, as well as maintaining health;” and finally “to limit calories, salt, sugar and (saturated) fat consistent with WHO guidelines” (4). National dietary guidelines increasingly recommend diversifying the types of protein consumed and increasing the share, diversity and sustainability of plant-based foods (21, 22), and this has been taken into account when setting the standards. It has been shown that transitioning toward more plant-based diets would reduce diet-related greenhouse gas emissions by 29–70 percent, and premature mortality by 6–10 percent (25).

In addition to plant-based ingredients, also dairy consumption is below recommendations in non-western regions. Dairy intake continues to be recommended in moderation by most dietary guidelines (3, 4, 22) as it is especially important for children’s development due to its beneficial role in growth and for bone health (26).

Based on the dietary guidelines for sustainable healthy plant-forward diets, ingredients included in the PNS are fruits, vegetables, legumes, nuts, seeds wholegrain and dairy.

##### 2.1.3.2 Macronutrients

Protein intakes are suboptimal globally with some regions overconsuming protein, while others lack sufficient protein in their daily intake. Protein plays an important role in child development and for health maintenance throughout the life cycle. Protein diversification is identified as a priority gap when moving to more plant-based diets (24, 27), hence protein was included in the Positive Nutrition Standards.

Another underconsumed macronutrient often specifically mentioned in dietary guidelines is fibre. Most populations have an average intake of only 10-15 g of fibre a day, far from the general recommendation of 25–30 grams a day (2). Vegans generally have adequate fibre intakes, but meat-eaters and vegetarians can be at risk of inadequacy (24). Therefore, fibre was considered a relevant nutrient to include in the standards.

Omega-3 fatty acid intake, and especially intake of α-linolenic acid has been shown to be lower in meat eaters but also vegetarians compared to vegans (24), and hence omega-3 fatty acids were included in the PNS.

##### 2.1.3.3 Micronutrients

According to the WHO report on Essential Nutrition Actions most prevalent micronutrient deficiencies include vitamin A, iron and zinc (23). However, also the need for iodine supplementation is addressed. In addition, a recent analysis has suggested that there are 372 million preschool-aged children (aged 6–59 months) and 1·2 billion non-pregnant women of reproductive age (aged 15–49 years) with one or more micronutrient deficiencies worldwide (17), confirming earlier estimations that from the total world population likely more than 2 billion people are undernourished (17). According to this analysis, deficiencies include vitamin A, vitamin B12, folate, iron and zinc. Therefore, all micronutrients mentioned were selected for inclusion in the PNS.

The selection of additional relevant micronutrients to be included in the PNS was primarily based on the recent comprehensive systematic review that investigated and compared nutrient intakes in meat-based, vegetarian and vegan diet pattens in different regions. The review showed intakes and status of calcium, iodine, iron, vitamin B12, vitamin D and zinc are shown to be generally lower in plant-based dietary patterns. On the other hand, people following plant-based diets, particularly vegan diets, had higher intakes of nutrients such as fibre, folate, vitamin C, vitamin E and magnesium, which were found to be at risk of inadequacy among meat-eaters (24). To ensure that the most common micronutrient inadequacies are tackled and to promote the benefits of moving toward a more plant-based diet while ensuring plant-based consumer nutritional needs are met, calcium, folate, vitamin C, vitamin E and magnesium were also included in the standards in addition to the five critical micronutrients.

Vitamin B2 was included as it is mainly present in animal foods and has been identified by Eat Lancet as a nutrient that may become critical when moving to a more plant-based diet (3). In addition, the recently launched Dietary Guidelines for Americans 2020–2025 consider potassium as a dietary component of general public health concern, and therefore potassium was also included in the PNS. In the guidelines vitamin C is mentioned as potential concern for children (28), justifying the inclusion of vitamin C. Together, the selected nutrients should cover most of inadequate nutrient intakes observed worldwide.

The aim of the PNS it is not to encourage food industry to start fortifying every product with one or more micronutrients. Both naturally present or added micronutrients via fortification are recognized if they meet the PNS defined standards. Fortification should only be considered on a case-by-case basis, to ensure that micronutrient levels will be effective and safe for the entire population (29).

##### 2.1.3.4 Overview ingredients and nutrients in scope

Considering the totality of scientific evidence and the guidelines to increase the intakes of plant-based foods, the Positive Nutrition Standards include the ingredients and nutrients as shown in Table 1.

**Table 1:** Overview of selected ingredients and nutrients.

Ingredients	Nutrients
FVNL (including fruits,vegetables legumes, funghi, nuts and seeds) Wholegrain Dairy (only for children’s products)	Protein Fibre Omega 3 fatty acids Vitamins: A, B2, B9 (folate), B12, C, D, E Minerals: Ca, Fe, I, K, Mg, Zn

#### 2.1.4 Rationale behind the standards and application to product groups

There is no clear guidance on how to set quantitative positive standards for food products. Therefore, the standards for positives nutrients were aligned with Codex Guidelines for Use of Nutrition and Health Claims (30). In absence of Codex guidance for ingredient content claims, standards for ingredients were based on international recommendations, and converted into product standards, considering the role in the diet, including appropriate serving size and frequency of consumption. More details are provided below and in the flowchart presented in the supplemental material.

Positive standards were set for all product groups and per product group the most relevant positives and quantities were selected considering their role in the diet. One exception is the ‘Animal Protein’ group, where no standards were defined to account for dietary recommendations to reduce intake of animal foods. However, it should be noted that in the other product groups, the protein and micronutrients coming from animal-based ingredients would still be recognized as positives.

##### 2.1.4.1 Standards for ingredients

Globally, there is not one aligned definition of fruits and vegetables. In addition, there are other plant-based ingredients that have nutritional benefits and are recommended by national dietary guidance, like fungi or nuts and seeds. Therefore, one standard FVNL (Fruits, Vegetables, Nuts & Legumes) was set to include plant-based ingredients like fruits, vegetables, legumes, fungi, nuts & seeds. A separate standard was set for grains to stimulate use of wholegrain in alignment with dietary guidance.

‘FVNL:’ (like for wholegrain and dairy) are in scope for all products groups, except Animal Protein. The standards are based on the WHO recommendation of 400 g or 5 portions of fruits and vegetables per day (15). This recommendation was translated into three different standards and applied to the different product groups considering the role of the product in the diet and appropriate serving size.

- 80 g/serve, corresponding to one portion of fruits & vegetables as defined by WHO (15). The standard is used for the product groups Main Meals, Plant Protein and Soups.
- 30 g/serve, which is about 1/3rd of a portion of fruits & vegetables. The standard is used for product groups that are consumed regularly as part of a meal, like Small Meals, Carbohydrate-based Dishes, Bread products, Cereals, Meal Sauces, hence these products are good vehicles for increasing FVNL intake. The standard is also used for Fruit & Vegetables Juices.
- 25% of the weight of the product (i.e., 25 g/100 g). This standard is used for all other product groups (see Table 2) for which meeting 80 g or 30 g per serving is not feasible.

**Table 2:** Overview of the positive nutrition standards.

Product group	Micronutrients (30)	FVNL (15)	Protein (30)	Fibre (30)	Wholegrain (31)	Dairy	Omega-3 (30)
Plant protein	15% RDA/serve	80 g/serve	10 g /100 g	3 g/100 g	NA	NA	NA
Soups			5 g /100 g				
Main meals					8 g/serve		
Small meals		30 g/serve					
Cereals							
Bread products							
Carbohydrate-based dishes							
Meal sauces					NA		
Fruit and vegetable Juices							
Mustards		25%					
All other products					8 g/serve		
Ice cream & desserts							
Snacks – sweet and savoury							
Kids ICE cream and desserts						25%	
Kids snacks – sweet and savoury							
Emulsion based sauces and cooking fats					NA	NA	0,3 g ALA/100 g
Spreads – sweet and savoury							NA
Water based sauces							
RTD and concentrated beverages							
Cereal and malt-based beverages							
Pickled and fermented vegetables			NA	NA			
Liquid bouillons and seasonings							
Universal bouillons and seasonings							
Dish specific bouillons and seasonings							
Toppings – sweet and savoury							
Cooking and baking agents							
Leaf teas, herbal infusions and coffee							
Animal protein	NA	NA	NA	NA	NA	NA	NA

Wholegrain: in absence of clear guidance from global health authorities, the standard was set at 8 g per serve as used by the Whole Grain Initiative (31), which can be regarded as significant amount and in line with the definition used in the US Dietary Guidelines (28). Wholegrain is in scope for all cereal/grain-based product groups and snacks that can contribute to the increase of wholegrain intakes.

Dairy: dairy is considered as a positive ingredient exclusively for children’s products (e.g., Kids Ice Creams and Kids Snacks – Sweet & Savoury) given dairy’s importance for children’s’ healthy growth and development. Similar to FVNL, the dairy standard was set at 25% of the weight of the product (i.e., 25 g/100 g).

##### 2.1.4.2 Standards for nutrients

The positive standards for nutrients follow international recommendations. The standards for positives are aligned with nutrition claims as set in the Codex Guidelines for Use of Nutrition and Health Claims (30). However, not all national regulations strictly follow Codex. Therefore, for Unilever’s major markets (Europe, United States and China) standards are adapted to meet local regulations where needed.

Protein: for most product groups the protein standard is aligned with the level needed to make a ‘source of’ claim as defined by Codex (30), i.e., 5 g/100 g. For the Plant Protein group the standard is aligned with the amount needed to make a ‘rich in’ protein claim, i.e., 10 g/100 g. In addition to Animal Protein, the product groups out of scope for protein are: Bouillons & Seasonings, Leaf Teas, Herbal Infusions & Coffee, Cooking & Baking Agents, Toppings – Sweet & Savoury. Despite the potential of these products to meet the amount needed for ‘source of’ when calculated per 100 g because of the inherent protein content, the serving size of the product ‘as sold’ is so low (e.g., for coffee 2 g and bouillons 5 g), that it would not be credible to count these as meaningful contributors of protein in the diet. Hence, they have been excluded.

Fibre: the standard is aligned with the amount needed to make as ‘Source of’ fibre claim as defined by Codex (30) at 3 g/100 g. The product groups out of scope for fibre are the same as for protein, following the same rational.

Omega-3 fatty acids: the standard is aligned with the level of α-linolenic acid (ALA) needed to make a ‘source of’ claim, i.e., 0.3 g ALA/100 g (30). Within the company’s portfolio, a standard for Omega 3 is only relevant for emulsion-based sauces, which can be good vehicles to help people increase their omega 3 intake.

Micronutrients: all micronutrient standards are aligned with the amount needed to make as ‘Source of’ claim as defined by Codex (30) which is 15% of the Nutrient Reference Value (NRV) per serve. All sources of micronutrients, inherently present or added by fortification are considered. Micronutrient standards were attributed for all products groups, except Animal Protein.

#### 2.1.5 Scoring of products

To assess if products meet the positive nutrition standards, products are scored as sold. This means only the ingredients and nutrients in the product as it is sold in store is counted, not the positives from other (fresh) ingredients added by consumers or chefs during preparation. For products containing fruits or vegetables in dried format, rehydration factors are applied when scoring. An overview of all food groups and positive standards applied is shown in Table 2.

### 2.2 Modeling

To evaluate the potential impact of our positive standards on population intakes, modeling was conducted using complete food consumption survey data. The modeling was performed using data from the US NHANES 2017–2018 survey (19), as the US survey was the only survey that had data available to calculate ingredient intakes in addition to nutrient intakes.

#### 2.2.1 Food consumption and composition data

The US NHANES 2017–2018 survey comprises different components. The What We Eat In America (WWEIA) 2017–2028 database is the dietary intake component of the survey, consisting of two 24-h recall data for a representative population sample of individuals ranged from birth up to 79 years. The Food and Nutrient Database for Dietary Studies (FNDDS) database provides the nutrient values for foods and beverages reported in WWEIA per 100 g (32). In addition, the Food Patterns Equivalents Database (FPED) includes different food patterns components, measured as cup equivalents of Fruit, Vegetables, and Dairy and ounce equivalents of Grains and Protein Foods (33). As the products in our portfolio are not designed for infants and toddlers, intake data for individuals ≥2 years were included in the modeling.

#### 2.2.2 Modeling scenario’s

The modeling was performed to the positive standards and product groups as mentioned in Table 3.

**Table 3:** Standards and product groups included in the modeling.

Positive	Standard	Food group
FVNL	80 g/serve	Main meals
	30 g/serve	Small meals Carbohydrate-based dishes Meal Sauces Soups
Wholegrain	8 g/serve	Main meals Small meals Carbohydrate-based dishes Bread products Cereals Snacks – sweet and savoury (kids and regular)

For all components two scenarios were analyzed:

- A baseline scenario: in which population intakes were calculated based on the original survey data
- A reformulation scenario: in which population intakes following ‘hypothetical re-formulation’ were calculated to meet the positive standards as defined for selected food groups.

For fibre two scenarios were analyzed: one applying the general standard based on Codex (30) and another applying the alternative standard following US regulation specifically (34).

#### 2.2.3 Food group alignment

The data of the FNDDS and FPED were merged into one single dataset. Then the food-groups included in these datasets were aligned with the product groups of the positive nutrition standards (Table 2), using the same product group reclassification and serving sizes as defined for a previous modeling exercise on the potential impact of standards for nutrients to limit (20). The US survey used a “foods-as-consumed” approach in which food codes correspond to mixed dishes in the dataset. This means that ingredients from dish recipes were not separated. For example, if a respondent reported consumption of ‘Spaghetti Bolognese’, the food was classified under the food group Main Meal. However, if a respondent reported consumption of spaghetti sauce and pasta as separate items, the items were categorized as Meal Sauces and Carbohydrate-based Dishes, respectively. Serving sizes were based on Unilever reference serving sizes where available (e.g., 30 g for Small Meals). For foods with more varying serving sizes (i.e., Snacks – Sweet and Savoury and Ice-Cream and Desserts), serving sizes were based on US specific data (35).

#### 2.2.4 Data preparation

After the food group alignment step, additional data preparation was required to perform the scenario analysis. The FPED measures vegetables in cup equivalents and wholegrain in ounce equivalents whereas the positive standards are set in grams. Therefore, these standards had to be converted to cups and oz. eq. respectively.

For FVNL:

- The WHO recommendation is 400 g per day for F&V (15), of which half was assumed for vegetables only
- The US recommendation for vegetables is 2.5 cup eq./day
- If standard is 80 g/serving: 80/200 = 40%, so 40% of 2.5 = 1 cup eq. serving
- If standard is 30 g/serving: 30/200 = 15%, so 15% of 2.5 = 0.375 cup eq./serving

For wholegrain: 1 g = 0.035 oz. eq., so 8 g/serving = 0.28 oz. eq./serving.

The FPED group vegetables includes subgroups of different vegetables, tomatoes, potatoes, other starchy vegetables, and beans and peas computed as vegetables (33). As the standard for FVNL does not include starchy vegetables, the variable for vegetables had to be redefined by excluding potatoes and other starchy vegetables. Although PNS includes one standard for FVNL, for the modeling only the redefined variable for vegetables (including beans and peas) was included as vegetables were felt more logical to increase in selected meals and dishes as opposed to fruits. Because in the FPED nuts and seeds are included under protein foods and measured in oz. equivalents instead of cups equivalents, nuts and seeds were also not included in the modeling.

As standards are expressed per serving and data in the FPED were provided per 100 g, values were re-calculated from per 100 g to per serving and benchmarked against the positive standards. In the reformulation scenario, values not meeting the standard were increased to the value of the standard. Finally, values were reconverted back from per serving to per 100 g and the updated values per 100 g were used to perform the reformulation scenario analysis.

#### 2.2.5 Data analysis

Scenario analysis was conducted using DaDiet Software of Dazult, Maynooth, Ireland (36). This web-based software tool allows accurate estimation of exposure to nutrients and to substances added to foods. To prepare the appropriate food composition dataset, we used JMP (version 14) and exported the file to excel for uploading in Dadiet.

The primary outcome for the analysis was the percentage change per target ingredient/nutrient in the reformulation scenario compared to the baseline scenario. Population mean nutrient intakes were calculated using ratio estimation and nonparametric techniques, incorporating survey weights to provide representative intakes for specific population groups. Outcome measures were calculated for the total diet and selected food groups. Mean population intakes at baseline and after hypothetical reformulation were plotted against the US recommended daily values for the general population: 2.5 cup eq. for vegetables, 3 oz. eq. for wholegrain and 28 g for fibre (28).

## 3 Results

**Figure 2:**
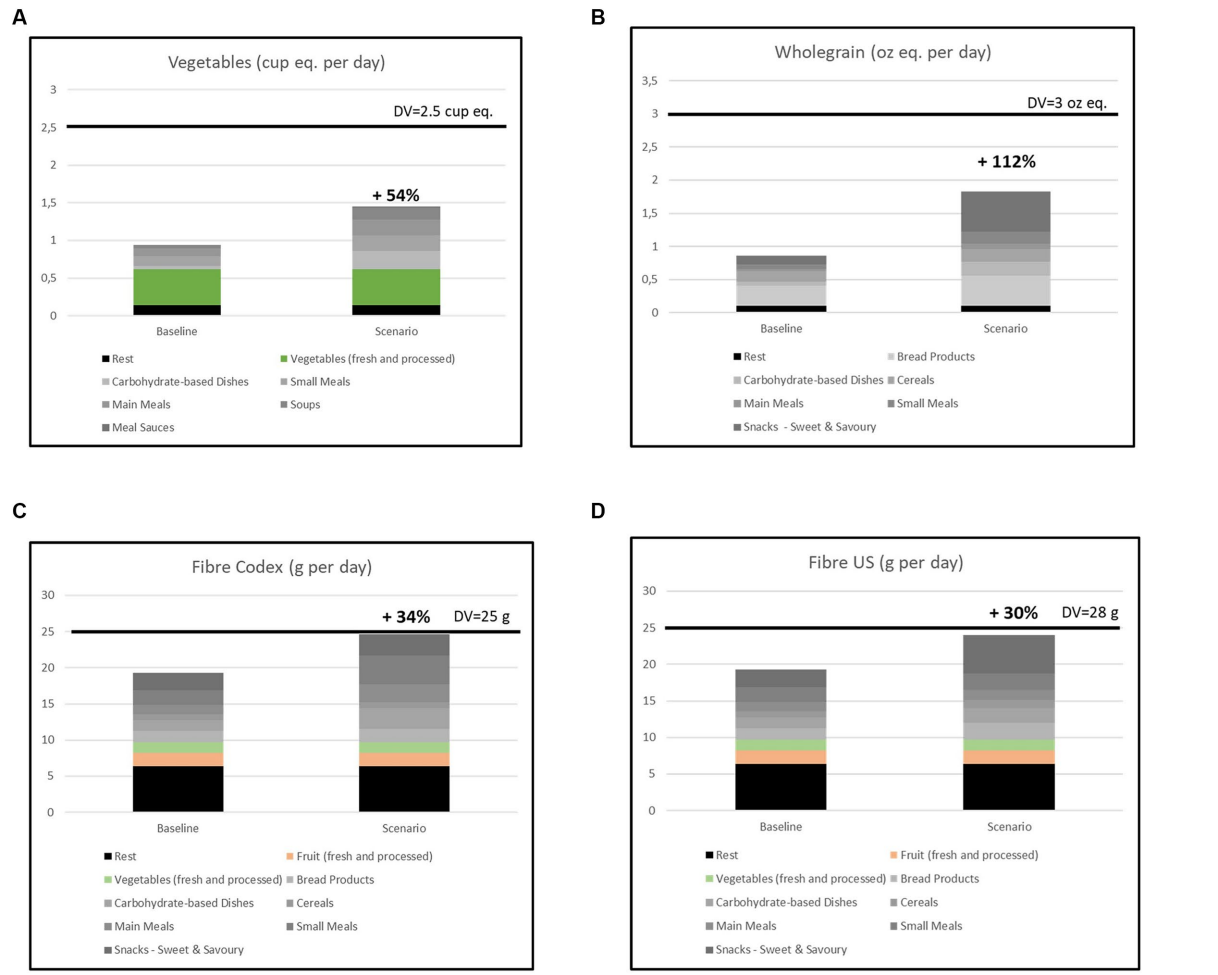
Mean population intakes at baseline and after hypothetical reformulation for **(A)** Vegetables, **(B)** Wholegrain **(C)** Fibre – Codex scenario, **(D)** Fibre – US scenario. The grey line represents the recommended daily value.

Results are presented in Figures 2A–D. Results show that reformulation of food products toward the positive standards would increase intakes of vegetables, wholegrain and fibre.

### 3.1 Vegetables

For vegetables the projected increase in mean population intake after hypothetical reformulation toward the standards was 54% (Figure 2A). As vegetables themselves also contribute to vegetable consumption, the graph also shows the specific contribution of the vegetables themselves. As there is no standard for these products in PNS the effect of vegetables themselves is the same in both scenarios. Most of the increase in vegetable intake was accounted for by increasing the vegetable content of carbohydrate-dishes (21%), but also increasing the amount of vegetables in main meals, small meals or soups are shown to help to increase vegetable intakes with about 10% per product group. However, the increase in the amount of vegetables in selected product groups would not be sufficient to meet the recommended daily intake for vegetables.

### 3.2 Wholegrain

For wholegrain the projected increase in mean population intake after hypothetical reformulation toward the standards was 112% (Figure 2B). Especially, increasing the amount of wholegrain in snacks increased wholegrain intakes (up to 54%), but also in carbohydrate-based dishes (19%), bread products (17%), small meals (13%) and to a lesser extent main meals (6%) and cereals (3%). Similar to vegetables, increasing the amount of wholegrain in the selected product groups would not be sufficient to meet the daily recommendation for wholegrain.

### 3.3 Fibre

For fibre the projected increase in mean population intake after hypothetical reformulation toward the standards was 34% using the Codex scenario with standards in g/100 g (Figure 2C). Although the PNS do not include standards for fruits and vegetables as separate product groups, these product groups are generally recognized sources of daily fibre intake. Therefore, the graph also shows the specific contribution of these two product groups. When applying the specific US scenario with standards in g/serving the increase would be slightly smaller with 30% (Figure 2B). The two scenarios differed when looking at the potential impact of application of the standards to the specific product groups. Using the Codex scenario, the biggest increase in fibre intake could be achieved via reformulation of small meals (13%), while for the US scenario the increase for small meals was only very small (1%). In the US scenario the biggest increase was shown for snacks (19%), whereas the increase for snacks in the Codex scenario was much smaller (4%). In both scenario’s intakes after hypothetical reformulation were close to the daily recommendation for fibre.

## 4 Discussion

Although in the past decades, the focus in nutrient profiling and product reformulation has been on reducing nutrients to limit, it is also important to consider how food reformulation and innovation can help to ensure or increase the amount of ingredients and nutrients with a positive impact on health. There is no clear guidance on how to set positive nutrition standards for food products. In addition, there is only a limited number of other nutrient profiles which include standards for positive nutrients and ingredients. The positive nutrition standards described in this paper were designed with the aim to increase dietary recommended nutrients and ingredients that people should eat more of, for human and planetary health.

The positive nutrition standards were set to be in line with regulations. Europe regulation is in line with Codex, but for important big markets like US and China, the standards allow for adaptation to local regulation. However, the modeling showed that differences in results observed for fibre where only minor when applying the Codex scenario with standards per 100 g vs. the US specific scenario with standards per serving.

Key strength of the positive nutrition standards is that ingredients and nutrients were selected in line with dietary guidelines and recommendations for a healthy and more plant-based diet. Daily recommendations and guidance for making nutrition claims were converted into product group specific standards for nutrients and ingredients, considering the role of the product group in the diet, including appropriate serving size and frequency of consumption.

Another strength is that, in contrast to many other nutrient profiling models, the potential impact of the positive nutrition standards was evaluated by modeling using actual food consumption data from the US NHANES. The modeling served as a proof of principle analysis and showed that reformulation of food products toward the standards for fruits & vegetables, wholegrain and fibre in selected product groups could substantially increase mean population intakes of these ingredients and nutrients.

It is acknowledged that modeling represents a theoretical approach with its limitations, and results are dependent on the quality of the input data and assumptions made. It reflects a best-case scenario, assuming all foods in selected product groups would meet the specific standards. It may not be entirely realistic for example for a traditionally meat-based soup to meet the standard for vegetables, or a chocolate-based snack to meet the standard for wholegrain or fibre. Nevertheless, the modeling shows the potential impact on intakes if people would move to more nutrient dense options.

One of the limitations is that only the US food consumption survey has data available on ingredient consumption, such as vegetables and wholegrain. Hence the impact of the ingredient standards could only be modeled for the US and not for a wider number of countries. The modeling were limited to vegetable, wholegrain and fibre. The protein standards were not included in the modeling because in the US protein intake is already in line with daily recommendations, and intake of Plant Protein products is still very low. The potential impact of the standards for micronutrients was also not included in the modeling. As stated earlier, the need for fortification needs to be considered on a case-by-case basis. This means there should be an acknowledged need for micronutrients or a clear public health problem by the target population, indicated by deficiencies and/or inadequate intakes (29). The US already applies health policies for staple fortification to address potential deficiencies, like mandatory fortification of cereals with folic acid. Although we did not model the impact of the standards for micronutrients for the US population, the potential impact of fortification of bouillons and seasonings with iron and iodine was evaluated in another study based on intake data of different Asian countries. The study showed that fortifying bouillons & seasonings with iron and iodine at levels in line with the PNS can help to increase the intake with approximately 33% of the RDA (37).

A third limitation is that a “foods-as-consumed” approach was used in the modeling, and ingredients from recipes were not separated. Therefore, the survey included a relatively high number of meals and dishes. Dishes were often mixtures of different components (meat, carbohydrates, vegetables) and it was not clear whether there was one clear main component making up over 70% of the meal or dish or if it was a mixture of components in more equal amounts. Decisions on classifications were made in alignment with the team involved in the modeling. However, we do not expect that this has had an impact on results and conclusions.

Despite the limitations, the modeling results provide a fair indication that applying the standards could help to substantially increase intake of positive ingredients and nutrients. However, results also show that reformulation of foods toward the positive nutrition standards alone may not always be sufficient to meet the daily values for daily intake for positive ingredients and nutrients. Therefore, next to reformulation and innovation, a multistakeholder approach is needed to ensure required dietary shifts by encouraging consumers to change their eating behaviors. Nevertheless, convenient and affordable processed foods with longer shelf life can be important in filling nutrients gaps in populations worldwide (18).

Within Unilever, which is one of the largest food manufacturers worldwide, the standards are used to encourage reformulation and innovation. In 2020, the company committed to double the number of products sold that deliver positive nutrition by 2025. Next to increasing the number of products delivering positive nutrition, the company also continues applying the product standards for nutrients to limit (20). To avoid the compensation of nutrients to limit by positive ingredients and nutrients standards for positives and nutrients to limit are separate sets. With both sets of standards, the company continues to improve the nutritional quality of the portfolio. With the PNS we hope to inspire other organizations to look beyond the nutrients to limit and include ingredients and nutrients to be encouraged in their reformulation targets or nutrition profiling methods to help consumers worldwide to shift to more healthy and sustainable diet.

## 5 Conclusion

The positive nutrition standards were set by translating WHO and Codex guidance into product group standards, considering the role of the product group in the diet, including appropriate serving size and frequency of consumption. The modeling results show that reformulation toward these standards would be impactful, moving intakes closer to recommendations. The standards may serve as a positive example in discussions on nutrient profiling and standard setting. However, next to product reformulation by food industry a multistakeholder approach is needed to encourage consumers to make additional dietary shifts that are required to meet the recommended daily values for positive nutrients and ingredients.

## Data availability statement

Publicly available datasets were analyzed in this study. This data can be found at: https://wwwn.cdc.gov/nchs/nhanes/continuousnhanes/default.aspx?BeginYear=2017.

## Ethics statement

The studies involving humans were approved by the NCHS Ethics Review Board, National Center for Health Statistics, Center for Disease Control and Prevention, US. The studies were conducted in accordance with the local legislation and institutional requirements. Written informed consent for participation was not required from the participants or the participants’ legal guardians/next of kin in accordance with the national legislation and institutional requirements.

## Author contributions

MD-K: Conceptualization, Data curation, Formal analysis, Investigation, Methodology, Project administration, Software, Validation, Writing – original draft. SC: Conceptualization, Data curation, Formal analysis, Investigation, Methodology, Project administration, Software, Validation, Visualization, Writing – original draft. CL: Conceptualization, Methodology, Resources, Supervision, Writing – review & editing. JW: Conceptualization, Funding acquisition, Resources, Supervision, Writing – review & editing.
