# Supplementary material for: Improving the nutrient quality of foods and beverages using product specific standards for positive nutrients and ingredients will help to increase mean population intakes toward dietary guidelines
Source: Front Nutr. 2023 Dec 6;10:1292231. doi: 10.3389/fnut.2023.1292231 (PMC10730655; doi:10.3389/fnut.2023.1292231)
Supplement: Supplementary file 1 [file Data_Sheet_1.PDF]

frontiers

Supplementary Material

Improving the nutrient quality of foods and beverages using product specific standards for positive nutrients & ingredients will help to increase mean population intakes towards dietary guidelines

Mariska Dötsch-Klerk[1*], Sara Carvalho [1*], Corrine F. Lawrence[1] and Julie I. Willems[1]

[1]Unilever Foods Innovation Centre, 6708 WH Wageningen, The Netherlands

*** Correspondence:**

Mariska Dötsch-Klerk: mariska.dotsch@unilever.com

Sara Carvalho: sara.carvalho@unilever.com

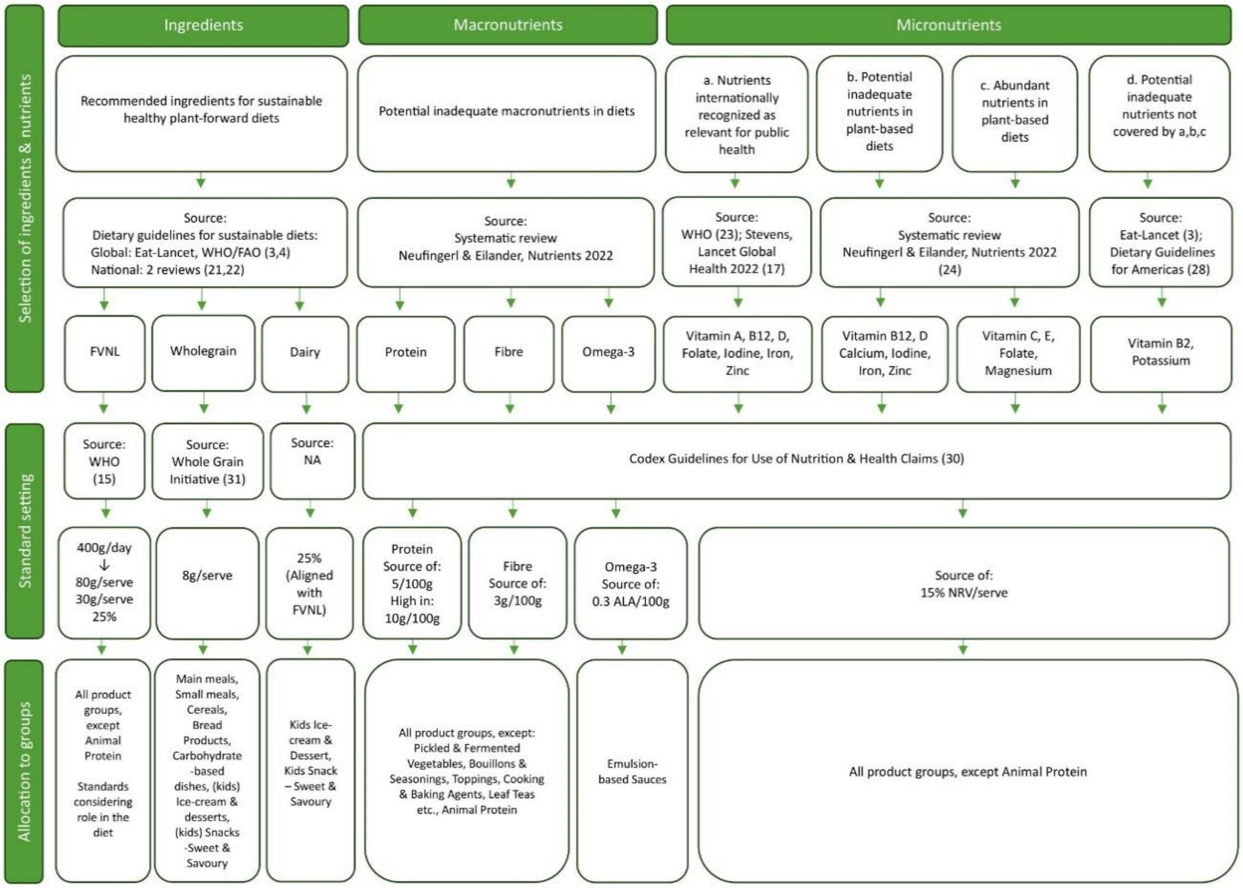

Supplementary Figure 1. Flowchart of selection of ingredients and nutrients, standard setting and product group allocation
